# Experiences, challenges and perspectives for ensuring end-of-life patient care: A national online survey with general practitioners in Germany

**DOI:** 10.1371/journal.pone.0254056

**Published:** 2021-07-27

**Authors:** Jannik M. Tielker, Jan P. Weber, Steffen T. Simon, Claudia Bausewein, Stephanie Stiel, Nils Schneider

**Affiliations:** 1 Institute for General Practice, Hannover Medical School, Hanover, Germany; 2 Department of Palliative Medicine, University Hospital of Cologne, Faculty of Medicine, University of Cologne, Cologne, Germany; 3 Department for Palliative Medicine, University Hospital Munich, Ludwig-Maximilians-Universität, Munich, Germany; University of the Pacific, UNITED STATES

## Abstract

**Background:**

The SARS-CoV-2 (COVID-19) pandemic is posing major challenges for health care systems. In Germany, one such challenge has been that adequate palliative care for the severely ill and dying (with and without COVID-19), as well as their loved ones, has not been available at all times and in all settings., the pandemic has underlined the significance of the contribution of general practitioners (GPs) to the care of severely ill and dying patients.

**Objectives:**

To describe GPs’ experiences, challenges and perspectives with respect to end-of-life care during the first peak of the pandemic (spring 2020) in Germany.

**Materials and methods:**

In November and December 2020, a link to an Unipark online survey was sent to GPs registered on nationwide distribution lists.

**Results:**

In total, 410 GPs responded; 61.5% indicated that the quality of their patients’ end-of-life care was maintained throughout the pandemic, 36.8% reported a decrease in quality compared to pre-pandemic times. Of the GPs who made home visits to severely ill and dying patients, 61.4% reported a stable number of visits, 28.5% reported fewer visits. 62.7% of the GPs reported increased telephone contact and reduced personal contact with patients; 36.1% offered video consultations in lieu of face-to-face contact. The GPs reported that relatives were restricted (48.5%) or prohibited from visiting (33.4%) patients in nursing homes. They observed a fear of loneliness among patients in nursing homes (91.9%), private homes (87.3%) and hospitals (86.1%).

**Conclusions:**

The present work provides insights into the pandemic management of GPs and supports the development of a national strategy for palliative care during a pandemic.

To effectively address end-of-life care, GPs and palliative care specialists should be involved in COVID-19 task forces on micro, meso and macro levels of health care.

## Introduction

### Background

The SARS-CoV-2 (COVID-19) pandemic is posing major challenges for health care systems across the world. Throughout the pandemic, the primary goal has been to protect the population from infection and provide medical care for infected persons. In the first peak of infections in spring 2020, the German federal government, in consultation with the federal states, enacted containment measures for the general public, including social distancing, the isolation of positive or suspected cases, a ban on admissions to nursing homes and a ban on visitation in hospitals, nursing homes and hospices [1–5]. Although these were helpful strategies to reduce infection and mortality [6, 7], by March 2021, more than 70,000 people had died from or with COVID-19 in Germany [8].

Throughout the pandemic, people have continued to require end-of-life care for cancer and other advanced chronic diseases. However, in Germany as all over the world [9], adequate palliative care for the severely ill and dying (with and without COVID-19), and their loved ones, has not been available at all times and in all settings during the pandemic [10, 11]. This has caused psychological, social and spiritual distress for patients, thereby compromising their quality of life.

Palliative care aims at maintaining patients’ quality of life [12, 13]. It can be provided on at least two levels: general and specialist [14, 15]. When administering palliative care, GPs maintain close contact with patients and their relatives. Frequently, they form significant and long-term relationships with patients, initiating and coordinating care and further treatment with other health care providers. Thus, in Germany, GPs represent a key provider of general palliative care [16, 17], and the COVID-19 pandemic has served to underline their significance in this respect [18, 19].

### Study aim

This paper aims at describing GPs’ experiences, challenges and perspectives relating to general palliative care during the COVID-19 pandemic in Germany.

## Materials and methods

The present study, based on an online survey with GPs in Germany, is part of the German collaborative project "National Strategy for Palliative Care of Severely Ill and Dying People and their Relatives in Pandemics (PallPan) in Germany," led by the National Research Network of University Medicine on COVID-19. PallPan aims at developing and achieving consensus on a national strategy for the care of seriously ill, dying and deceased adults (with and without COVID-19) and their relatives during a pandemic.

### Pre-study

Recent subjective field experiences were explored through two informal conversations with resident GPs in July and August 2020. The topics that emerged in these conversations were further discussed and explored in September 2020 within an online focus group involving three GPs, as well as telephone interviews with two GPs. The focus group and interviews were audio-recorded and transcribed verbatim. Main reported experiences and challenges were for instance limited home visits, restricted physical closeness to patients and less visits from relatives, less body-related therapies, and an increased isolation of patients.

### Survey development and pre-test

Between September and November 2020, a standardized questionnaire was developed using the synthesized findings from our pre-study. The questionnaire was pre-tested by six GPs using the online survey tool Unipark, with special attention paid to the questionnaire’s structure and coherence, comprehensibility, technical aspects and duration.

Information was collected on the following sections:

1. sociodemographic data on the study population;
2. patient contact;
3. telephone contact;
4. video consultation;
5. cooperation with other health care providers;
6. psychosocial aspects; and
7. needs and suggestions for managing end-of-life care in the context of a pandemic.

The questionnaire used 4- and 5-point verbal rating scales (i.e. *totally agree*, *rather agree*, *rather disagree*, *fully disagree*) to determine the extent of (dis)agreement with the presented statements, which reflected subjective experiences, challenges and perspectives pertaining to end-of-life care during COVID-19. Free-text options were also provided to allow for respondents’ comments on their individual provided statements.

### Recruitment of the study population

In November and December 2020, the information and invitation letter, including a direct, non-personalized link to the GP survey in Unipark, was sent to:

1. nine university institutes for general practice in seven federal states (Mecklenburg-Western Pomerania, Berlin, Lower Saxony, Hessen, North Rhine-Westphalia, Baden-Wuerttemberg, Bavaria), for distribution to their teaching and research networks;
2. three GP Associations in Lower Saxony and Bremen;
3. the German College of General Practitioners and Family Physicians;
4. the German Association for Palliative Medicine; and
5. the Competence and GP Training Center of Lower Saxony, for distribution to their members.

The research team had no direct access to the distribution lists of the abovementioned parties and cannot quantify the number of GPs who were contacted. However, we estimate that the survey was distributed to at least 3,000 GPs. In the invitation letter, participants were asked to forward the letter to other interested parties, thereby triggering a snowball effect to maximize the study population. Each participant was asked to complete the questionnaire only once. Participation was completely anonymous. The survey was open from November 23 to December 18, 2020.

### Data analysis

The SPSS 26 statistical software package was used to calculate descriptive statistics (mean value, standard deviation, minimum, maximum) and the absolute and percentage frequencies of the questionnaire data. Outliers were treated with the full dataset. Missing data are reported explicitly.

The qualitative analysis of the pre-study data and free-text comments was based on content analysis (according to Kuckartz), using MAXQDA version 18 [20]. The main categories of the qualitative interview guide were used as the basis for the questionnaire content domains.

### Ethical requirements

A written positive ethics vote (No. 9232_BO_K_2020 of 24.07.2020) for the project was issued by the Ethics Committee of the Hannover Medical School.

Prior to a subject’s participation in the online questionnaire, the participants had to confirm a check box that they have read and understood the written informed consent form concerning ethics and data protection and accept the regulations. Without this confirmation the participation was not possible.

## Results

### Sociodemographic data on the study population

The survey was completed by 410 GPs, comprising an approximately equal number of women and men. Their average age was 54 years (range 31–73 years), and they represented all 16 federal states in Germany. Approximately half of the GPs (51.5%) had completed additional training in palliative care. On average, they required 23 minutes to complete the questionnaire. Almost all of the GPs were experienced palliative care providers and reported that they had seen patients with COVID-19 in their practice (Table 1).

**Table 1:** Study population.

Variable	Answer options			
		M ± SD	Min–Max	N
Age (years)		53.7 ± 8.7	31–73	410
		%	n	
Gender	male	49.0	201	
	female	48.0	197	
	missing data	2.9	12	
Federal state	Baden-Wuerttemberg	8.8	36	
	Bavaria	16.1	66	
	Berlin	3.7	15	
	Brandenburg	1.2	5	
	Bremen	2.0	8	
	Hamburg	0.7	3	
	Hessen	7.3	30	
	Lower Saxony	40.7	167	
	Mecklenburg-Western Pomerania	2.4	10	
	North Rhine-Westphalia	7.3	30	
	Rhineland Palatinate	2.0	8	
	Saarland	0.2	1	
	Saxony	1.0	4	
	Saxony-Anhalt	0.7	3	
	Schleswig-Holstein	1.7	7	
	Thuringia	3.4	14	
Population figure	<5,000 inhabitants	21.0	86	
	5,000–20,000 inhabitants	35.4	145	
	20,000–100,000 inhabitants	21.0	86	
	>100,000 inhabitants	22.0	90	
	missing data	0.7	3	
Kind of medical practice	single practice	39.5	162	
	group practice or practice community	56.3	231	
	medical care center	3.2	13	
	missing data	1.0	4	
Additional training in palliative care	yes	51.5	211	
	no	48.0	197	
	missing data	0.5	2	
Cared for severely ill and dying patients (completed and ongoing) in 2020	none	1.2	5	
	1–10	23.2	95	
	11–50	54.4	233	
	51–100	12.4	51	
	>100	8.5	35	
	missing data	0.2	1	
Number of COVID-19 positive patients in practice in 2020	none	1.2	5	
	1–10	21.0	86	
	11–50	57.1	234	
	> 50	20.7	85	
		M ± SD	Min–Max	
Severe course of infection (e.g. inpatient hospital treatment)	number	3.2 ± 5.2	0–50	405
Died with severe course of infection	number	1.1 ± 3.0	0–40	

M = mean; SD = standard deviation; Min = minimum; Max = maximum; n = sub-sample; N = total sample; deviations to 100% possible due to rounding differences.

### Experiences and challenges during the pandemic

The following results for patient contact, telephone contact, video consultation, cooperation with other health care providers, psychosocial aspects, and needs and suggestions for end-of-life care in the context of a pandemic refer exclusively to GPs’ experiences caring for severely ill and dying patients during the first peak of the pandemic, in spring 2020.

#### Patient contact

The majority of respondents assessed the quality of their patients’ end-of-life care as consistent (61.5%), while 36.8% reported a decrease in quality relative to the pre-pandemic period. Of the GPs who made private home visits to severely ill and dying patients, 61.4% reported a consistent number of visits, 28.5% fewer home visits, and 9.1% more home visits (1.1% missing data) compared to the pre-pandemic situation. Similar results were found with respect to nursing home visits (Table 2). The most frequently cited reason for reduced visits was restricted access.

**Table 2:** Patient contact, telephone contact and video consultation.

Variable	Answer options	%	n	N
**Patient contact**				
Change in frequency of contact with severely ill and dying patients	yes, greater	7.1	29	410
	no, unchanged	55.1	226	
	yes, reduced	37.3	153	
	missing data	0.5	2	
Change in quality of end-of-life care	yes, improved	1.2	5	
	no, constant	61.5	252	
	yes, worsened	36.8	151	
	missing data	0.4	2	
Home visits to severely ill and dying patients	yes	94.1	386	
	no	5.1	21	
	missing data	0.7	3	
Change in frequency of private home visits to severely ill and dying patients	yes, greater	9.1	35	386
	no, unchanged	61.4	237	
	yes, reduced	28.5	110	
	missing data	1.1	4	
Change in frequency of nursing home visits to severely ill and dying patients in nursing homes	yes, greater	7.3	28	
	no, unchanged	48.2	186	
	yes, reduced	42.0	162	
	missing data	2.6	10	
**Telephone contact**				
Increased frequency of telephone versus personal contact with severely ill and dying patients	yes	62.7	257	410
	no	36.3	149	
	missing data	1.0	4	
Change in quality of end-of-life care for severely ill and dying patients receiving telephone rather than personal contact	yes, improved	2.3	6	257
	no, constant	56.8	146	
	yes, worsened	36.2	93	
	missing data	4.7	12	
Increased frequency of telephone versus personal contact with relatives of severely ill and dying patients	yes	69.0	283	410
	no	27.1	111	
	missing data	3.9	16	
Change in quality of support for relatives of severely ill and dying patients receiving telephone rather than personal contact	yes, improved	8.8	25	283
	no, constant	60.1	170	
	yes, worsened	26.1	74	
	missing data	5.0	14	
Accessibility for severely ill and dying patients outside practice opening hours	always available	41.7	171	410
	available at certain times (e.g. weekends)	31.7	130	
	not available	18.3	75	
	missing data	8.3	34	
**Video consultation**				
Video consultation offered in lieu of face-to-face contact	yes	36.1	148	410
	no	62.4	256	
	missing data	1.4	6	
Video consultation offered for severely ill and dying patients in their private homes	yes, on a regular basis	1.4	2	148
	yes, in individual cases	22.3	33	
	no, not at all	75.7	112	
	missing data	0.7	1	
Video consultation offered for severely ill and dying patients in nursing homes	yes, on a regular basis	2.7	4	
	yes, in individual cases	16.2	24	
	no, not at all	79.1	117	
	missing data	2.1	3	
Video consultation offered for relatives of severely ill and dying patients	yes, on a regular basis	0.7	1	
	yes, in individual cases	19.6	29	
	no, not at all	75.0	111	
	missing data	4.7	7	

n = sub-sample; N = total sample; deviations to 100% possible due to rounding differences.

#### Telephone contact

In total, 62.7% of the GPs reported increased telephone contact to replace personal contact with severely ill and dying patients. Of these, 36.2% indicated that the quality of end-of-life care worsened due to the lack of personal contact, because there were no physical examinations, communication was challenged and they were less able to provide emotional support. Similar problems were reported for telephone contact with relatives (Table 2).

#### Video consultation

In total, 36.1% of the GPs offered video consultation in lieu of face-to-face contact with severely ill and dying patients and their relatives, which was generally only realized in individual cases by primary care physicians at private homes and nursing homes. Many GPs stated that video consultation was not used or requested by severely ill and dying patients. Their cited reasons for this included technical difficulties, lack of user competence on the part of the patient and poor quality of care. Video consultation was, however, used to support relatives of severely ill and dying patients (Table 2).

#### Cooperation with other health care providers

The respondents rated their cooperation with other GPs, community nursing services and nursing homes as good. In contrast, they evaluated their cooperation with physio, occupational and other therapeutic professions, medical specialists, hospitals and health authorities as satisfactory, and thus slightly worse. Local health authorities, described as overburdened, were criticized primarily for their lack of accessibility.

The GPs described nursing homes’ hygiene concepts as inconsistent. In the free-text fields, some GPs (n = 15) reported that they were challenged in their attempts to access nursing homes. The main reason for this was that nursing home stakeholders were uncertain of how to interpret and apply hygiene-related contact restrictions. In addition, the GPs also reported problems admitting severely ill patients to nursing homes.

According to the GPs evaluation, many relatives could have been restricted (48.5%) or prohibited from visiting (33.4%) patients in nursing homes. The GPs perceived deterioration in the physical and mental health of patients in private homes and nursing homes as a consequence of this restricted contact (Table 3).

**Table 3:** Cooperation with other health care providers.

Variable		Answer options			
			M ± SD	Min–Max	N
Cooperation with outpatient nursing services		outpatient nursing services	2.0 ± 0.9	1–6	410
		other GPs	2.2 ± 1.1		
		nursing homes	2.4 ± 1.1		
		therapeutic professions	2.8 ± 1.2		
		medical specialists	2.9 ± 1.2		
		hospitals	3.0 ± 1.2		
		health care authorities	3.6 ± 1.5		
			%	n	
Problems with admitting severely ill and dying patients to nursing homes		quarantine required	42.9	176	
		neg. COVID-19 test required	56.8	233	
		long wait period	29.5	121	
		no problems	13.2	54	
		missing data	6.6	27	
Consistent hygiene concepts amongst nursing homes		yes	12.0	49	
		no	79.3	325	
		missing data	8.7	36	
Visitation of relatives permitted for severely ill and dying patients in nursing homes		yes	16.3	67	
		in part	48.5	199	
		no	33.4	137	
		missing data	1.7	7	
Change in physical health of severely ill and dying patients as a result of contact restriction	in private homes	yes, improved	0.7	3	
		no, constant	46.3	190	
		yes, worsened	43.7	179	
		missing data	9.3	38	
	in nursing homes	yes, improved	2.0	8	
		no, constant	34.6	142	
		yes, worsened	55.9	229	
		missing data	7.6	31	
Change in mental health of severely ill and dying patients as a result of contact restriction	in private homes	yes, improved	0.0	0	
		no, constant	15.9	65	
		yes, worsened	78.0	320	
		missing data	6.1	25	
	in nursing homes	yes, improved	0.2	1	
		no, constant	5.6	23	
		yes, worsened	89.5	367	
		missing data	4.6	19	

M = mean; SD = standard deviation; Min = minimum; Max = maximum; n = sub-sample; N = total sample; deviations to 100% possible due to rounding differences.

The GPs also perceived that relatives saying goodbye to their loved ones was only possible to a very limited extent (91.7%) or not at all (56.1%) (Table 4).

**Table 4:** Psychosocial aspects of severely ill and dying patients and their relatives.

Item GPs rating on:		Answer options									
		true		rather true		rather not true		not true		missing data	
		%	n	%	n	%	n	%	n	%	n
Expressed fear of severely ill and dying patients	COVID-19 infection	25.6	105	19.3	79	32.9	135	21.0	86	1.2	5
	loneliness in private homes	54.4	223	32.9	135	8.8	36	3.4	14	0.5	2
	loneliness in nursing homes	64.1	263	27.8	114	4.1	17	2.0	8	1.9	8
	loneliness in hospitals	65.9	270	20.2	83	4.1	17	1.0	4	8.8	36
	lower priority care in the event of hospitalization	28.3	116	28.0	115	24.1	99	12.0	49	7.6	31
	overtreatment in the event of hospitalization.	4.4	18	8.3	34	45.4	186	35.9	147	6.1	25
Relatives receive less information about patients due to contact restriction		46.6	191	39.3	161	8.8	36	3.2	13	2.2	9
Relatives find it stressful that they cannot support patients with their physical presence		85.6	351	13.7	56	0.2	1	0.2	1	0.2	1
Relatives limited in their ability to say goodbye to dying patients due to contact restriction		63.2	259	28.5	117	6.1	25	1.2	5	0.9	4
Relatives unable to say goodbye to dying patients due to contact restriction		19.5	80	36.6	150	26.3	108	14.6	60	2.9	12

n = sub-sample (total sample = 410); deviations to 100% possible due to rounding differences.

#### Psychosocial aspects of severely ill and dying patients and their relatives

The GPs observed an increased fear of loneliness among severely ill and dying patients in nursing homes (91.9%), private homes (87.3%) and hospitals (86.1%). With regard to the psychosocial burden on relatives, the majority of the GPs reported increased distress due to relatives’ reduced receipt of information about the patient (85.9%) and inability to support them with their physical presence (99.3%) (Table 4).

#### Needs and suggestions for end-of-life care in the context of a pandemic

The GPs identified social contact with relatives and face-to-face contact with physicians as the most important aspects of patient care (Table 5).

**Table 5:** Most important aspects of palliative patient care in pandemic times (up to three free text comments per person possible).

Categories	Given answers (n)	
Medical care	• maintaining face-to-face contact between physicians and patients (66) • offering professional care for patients (57) • having a stock of protective equipment for staff members (54) • maintaining home visits (32) • maintaining exchange and cooperation with colleagues (24) • offering advance care planning (22) • providing home care (18) • integrating specialized outpatient palliative care (18) • applying pain management (17) • setting no restriction of treatment (16)	• ensuring GP accessibility (15) • offering medical care (13) • maintaining exchange with caregivers (11) • offering enough time (11) • having professional expertise (10) • ensuring continuity of care (10) • enabling admission to hospital (9) • supporting relatives (8) • ensuring reliability (8) • paying attention to self-protection (8)
Psychosocial care	• enabling social contact, especially with relatives (154) • having contact between physicians and relatives (50) • ensuring psychosocial conversations with physicians (48)	• offering physical presence and closeness (17) • allowing physical touch (9) • integrating pastoral care (7)
Health policy	• promoting consistent regulation (20) • having hygiene concept (19) • engaging in good communication (16) • optimising COVID-19 testing (14)	• considering palliative care situations (12) • finding individual solutions (9) • providing sufficient nursing staff (7) • reducing bureaucracy (6)

n = sub-sample (total sample = 410).

In total, 92.4% of the GPs (fully/rather) agreed that GPs should be involved in local crisis teams, and 79.5% (fully/rather) agreed that palliative care physicians should also be involved. These local crisis teams were imagined to improve the exchange between outpatient and inpatient care providers and facilitate efficient, decentralized coordination and decision making at the local level.

## Discussion

The present study administered a nationwide online survey to collect GPs’ experiences, challenges and perspectives with respect to caring for severely ill and dying patients and their relatives during the COVID-19 pandemic. Almost all of the participating GPs had treated patients with COVID-19 in their practice. Throughout the pandemic, despite many efforts to adapt their individual practice management [21, 22], the GPs felt challenged in their ability to administer high quality palliative care.

Of note, the GPs reported deterioration in patients’ physical and mental health in both private and nursing homes, due to contact restriction. This concerning trend has also been observed by the German Association for Palliative Medicine and other professional organizations [11, 23–25].

In the present study, the GPs reported an increased fear of loneliness in their patients, as well as greater psychological distress in patients’ relatives, due to an inability to support their loved ones in person or to say goodbye. Girum et al., in a systematic review of 22 studies, demonstrated that quarantine and isolation measures have been effective in controlling the spread of COVID-19 [6, 7]. Thus, protective measures (e.g. social distancing) are recommended, especially for those at greater risk of infection [25]. However, while these measures may be important for managing the wider spread of the pandemic, the present study and other research has highlighted their serious physical and psychological consequences for severely ill and dying patients [26, 27]. To prevent these negative consequences, the GPs in our study recommended that social contact be maintained for patients receiving palliative care. The long-term effects of contact restriction and isolation on vulnerable groups must be investigated in future studies [28].

Germany’s Federal Government Commissioner for Long-Term Care and the Federal Minister of Health addressed this challenge in December 2020, proposing regulations for visitation to care facilities [29]. They emphasized the central role of social relationships for residents and listed basic measures to enable relatives to safely visit during the pandemic, with as few restrictions as possible.

In contrast, the German College of General Practitioners and Family Physicians advised, in its “Action Recommendation on the New Coronavirus,” reduced face-to-face contact between GPs and nursing home residents, where possible [30]. A reduction in home visits across both private and nursing homes was confirmed by approximately one-third of our GP respondents.

In the event of reduced home visits, the German College of General Practitioners and Family Physicians recommended that GPs should reduce personal patient contacts, but engage in telephone and video consultation [30]. They also recommended that these methods be widely applied in GP practices, to ease pressure [30]. Our survey showed that this occurred in almost two-thirds of our respondents’ practices, with the GPs increasing the frequency of their telephone consultations with patients relative to the pre-pandemic period. Saint-Lary et al. showed a similar trend for increased use of telephone communication with patients in their observational survey with French GPs [31].

In the present study, video consultation was offered by slightly more than one-third of the GP practices, for individual treatment. In contrast, another study found that 81% of GPs in Norway offered video consultation and found it suitable for maintaining patients’ continuity of care [32]. In our study, the GPs connected their minimal use of video consultation to their reservations about technical implementation, user competence and reduced quality of care (due to a lack of physical examination). Similar attitudes were found by Randhawa et al., in their qualitative study of 12 GPs in London [33]. Hawley et al. and Lieneck et al. reported further barriers to the use of this technology, including uncertainty among patients, concerns over data protection and lack of access to mobile devices among older patients [34, 35].

While reservations towards video consultation should not exclude the expansion of digital communication in health care, they reveal the need for training, broader implementation in nursing homes and clarity around data protection. In its draft “Digital Care and Nursing Modernization Act” of January 20, 2021, the Federal Cabinet addressed some of the abovementioned technical and infrastructure issues [36, 37]. As further guidelines on digital communication are developed, they must consider the views and experiences of health care providers, patients and their relatives [33, 35].

Based on their research in Atlanta, Kuntz et al. see great potential for video communication with relatives of palliative care patients. Drawing on data from their online survey with 67 caregivers and 10 semi-structured telephone interviews, the authors evaluate digitally-mediated family meetings as feasible and efficient. In addition, they conclude that video meetings might allow relatives to understand both the health and the care of the patient and to express their thoughts and feelings [38].

### Limitations

As we did not have access to the distribution lists used for our survey, we cannot comment on the response rate or non-responder characteristics, and we cannot fully exclude the possibility that some individual GPs participated in the survey more than once. Furthermore, due to the cross-sectional study design, we can only provide data related to changes over time during the pandemic. Since this survey was based on the GPs’ recall of their experiences, there is the potential for recall and confirmation bias. Finally, it can be assumed that, among the study participants, GPs with a particular interest in palliative care were disproportionately represented. Therefore these findings may not be fully representative of primary palliative care and end of life care provision by GPs across Germany.

## Conclusion

The present work provides insights into the nationwide pandemic management of a representative group of GPs in Germany. The findings may support the development of a national strategy for palliative care during a pandemic. We conclude that, during a pandemic, the preservation of face-to-face visits by relatives and the development of feasible and safe video communication should be prioritized. Finally, to address end-of-life care issues appropriately, GPs and palliative care specialists should be involved in COVID-19 task forces on the micro, meso, and macro levels of health care.
